# GeneValidator: identify problems with protein-coding gene predictions

**DOI:** 10.1093/bioinformatics/btw015

**Published:** 2016-01-18

**Authors:** Monica-Andreea Drăgan, Ismail Moghul, Anurag Priyam, Claudio Bustos, Yannick Wurm

**Affiliations:** ^1^Department of Computer Science, ETH Zürich, Zürich, Switzerland,; ^2^School of Biological and Chemical Sciences, Queen Mary University of London, London, UK and; ^3^Departamento de Psiquiatría y Salud Mental, University of Concepción, Concepción, Chile

## Abstract

**Summary**: Genomes of emerging model organisms are now being sequenced at very low cost. However, obtaining accurate gene predictions remains challenging: even the best gene prediction algorithms make substantial errors and can jeopardize subsequent analyses. Therefore, many predicted genes must be time-consumingly visually inspected and manually curated. We developed GeneValidator (GV) to automatically identify problematic gene predictions and to aid manual curation. For each gene, GV performs multiple analyses based on comparisons to gene sequences from large databases. The resulting report identifies problematic gene predictions and includes extensive statistics and graphs for each prediction to guide manual curation efforts. GV thus accelerates and enhances the work of biocurators and researchers who need accurate gene predictions from newly sequenced genomes.

**Availability and implementation**: GV can be used through a web interface or in the command-line. GV is open-source (AGPL), available at https://wurmlab.github.io/tools/genevalidator.

**Contact**: y.wurm@qmul.ac.uk

**Supplementary information:** Supplementary data are available at *Bioinformatics* online.

## 1 Introduction

The plummeting costs of DNA sequencing (Wetterstrand, 2015) have made *de novo* genome sequencing accessible to individual laboratories and even researchers (Nygaard and Wurm, 2015). However, identifying genes in a newly assembled genome remains challenging. Traditional gene prediction approaches involve either *ab initio* prediction via modelling of coding versus non-coding sequence or similarity-based prediction using independent sources. Relevant sources include protein-coding sequences from other organisms, or peptide or transcriptome sequences from the organism being studied. Modern algorithms combine both approaches (Cantarel *et al.*, 2008; Korf, 2004; Stanke *et al.*, 2008). The recent ability of obtaining large amounts of RNA sequences at low cost (Hou *et al.*, 2015) has led to a dramatic improvement in the performance of similarity-based algorithms and thus gene prediction quality (Goodswen *et al.*, 2012) albeit only for expressed genes. Despite this, the accuracy of gene prediction tools (e.g. Alioto *et al.*, 2013; Cantarel *et al.*, 2008; Keller *et al.*, 2011; Lomsadze *et al.*, 2014; Wilkerson *et al.*, 2006) remains disappointing (Yandell and Ence, 2012). Typical errors include missing exons, non-coding sequence retention in exons, fragmenting genes and merging neighboring genes. Automated gene prediction quality evaluation tools analyze exon boundaries (Eilbeck *et al.*, 2009; Yandell and Ence, 2012) or focus on subsets of highly conserved genes (Parra *et al.*, 2007). Unfortunately, such tools ignore most of the information present in frequently updated databases such as SwissProt or Genbank NR. Visual analysis is thus required to identify errors and manual curation is needed to fix them. This requires tens of minutes to days for one gene (Howe *et al.*, 2008) – a daunting task when considering analyses of dozens of species each with thousands of genes (Pray, 2008; Simola *et al.*, 2013).

We thus created GeneValidator (GV), a tool to evaluate quality of protein-coding gene predictions based on comparisons with similar known proteins from public and private databases. GV provides quality evaluations in text formats for automated analysis and in highly visual formats for inspection by researchers.

## 2 Approach

**Fig. 1.**
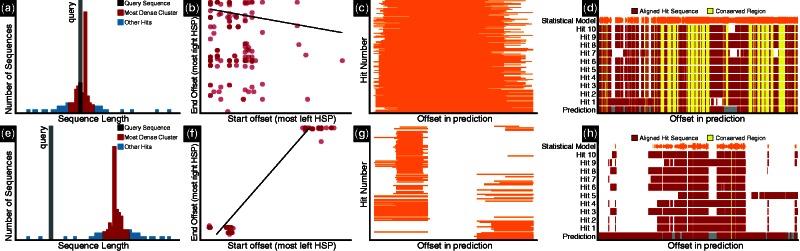
Contrasting GV graphs: (**a**), (**e**) sequence lengths; (**b**), (**f**) HSP offsets; (**c**), (**g**) overviews of hit regions; (**d**), (**h**) conserved regions. Graphs (a–d) were produced with a sequence for which GV detected no problems. The other graphs show typical problems: (e) query is short; (f), (g) query sequence is a fusion of unrelated genes; (h): query includes sequence absent from first 10 hits

For each new gene prediction, BLAST (Camacho *et al.*, 2009) identifies similar sequences in Swiss-Prot (The UniProt Consortium, 2014), Genbank NR (Benson *et al.*, 2010) or other relevant databases. Subsequently, GV performs up to seven comparisons between the gene prediction and the most highly significant hit sequences or high-scoring segment pairs (HSPs). The results of each comparison indicate whether characteristics of the query gene prediction deviate from those of hit sequences. The following four comparisons are performed on all queries:

**Length**: We compare the length of the query sequence to the lengths of the most significant BLAST hits using hierarchical clustering (Fig. 1a, e) and a rank test. A particularly low or high rank can suggest that the query is too short or too long.

**Coverage**: We determine whether hit regions match the query sequence more than once using a Wilcoxon test. Significance suggests that the query includes duplicated regions (e.g. resulting from merging of tandem gene duplicates).

**Conserved regions**: We align the query to a position specific scoring matrix profile derived from a multiple alignment of the ten most significant BLAST hits. This identifies potentially missing or extra regions (Fig. 1d, h and Supplementary Fig. S2).

**Different genes**: Deviation from unimodality of HSP start and stop coordinates indicates that HSPs map to multiple regions of the query. If this is the case, we perform a linear regression between HSP start and stop coordinates, weighting data points proportionally to BLAST significance (see Fig. 1b, c, f, g). Regression slopes between 0.4 and 1.2 (empirically chosen values) suggest that the query prediction combines two different genes (see Supplementary Fig. S1).

Two additional analyses are performed on nucleotide queries:

***Ab initio* Open Reading Frame (ORF)**: We expect a single major ORF. Frameshifts, retained introns or merged genes can lead to presence of multiple major ORFs.

**Similarity-based ORFs**: We expect all BLAST hits to align within a single ORF. This test is more sensitive than the previous when a query has HSPs in multiple reading frames.

An additional analysis is performed for MAKER gene predictions:

**MAKER RNASeq Quality Index**: MAKER gene predictions include a quality index (in the FASTA defline) indicating the extent to which the prediction is supported by RNAseq evidence. GV considers this information when it is available.

Each analysis of each query returns a binary result (i.e. similar or different to BLAST hits) according to a *P*-value or an empirically determined cutoff. The results for each query are combined into an indicative overall quality score from 0 to 100. The scores allow comparing overall qualities of different gene sets, or identifying the highest- or lowest-quality gene predictions within a gene set.

The individual and global scores are provided in JSON and tab-delimited text formats, and as an HTML report that can be viewed in a web browser (Supplementary Fig. S3). Importantly, this HTML report includes up to five graphs for each gene (Fig. 1), as well as explanations of the analyses and results. These visualizations can be particularly useful to biocurators improving gene predictions.

## 3 Usage

GV is installed as a ruby gem (Bonnal *et al.*, 2012). The user provides FASTA protein or nucleotide gene predictions; BLAST is run remotely (NCBI) or on a local database, or the user provides an existing BLAST output. Alternatively, a web wrapper provides an elegant graphical interface and a programmatic jQuery API. Finally, GV can already be used from within the Afra genome annotation editor (Priyam *et al*. unpublished).

## 4 Discussion

GV’s power comes from leveraging large, frequently-updated databases, using multiple metrics, input/output format flexibility and importantly its multiple data visualization approaches. Indeed, visualization is crucial for understanding genomic comparisons (Nielsen *et al.*, 2010; Riba-Grognuz *et al.*, 2011).

The code underlying GV respects best practices in scientific software development (Wurm, 2015). However, GV’s analyses depend on BLAST-identification of homologs in databases which include low-quality sequences, on expecting similar gene sequence and structure among homologs, and on empirically chosen cutoffs. Binary results of individual tests are thus indicative rather than infallible. Similarly, GV’s overall quality evaluations are not ground truths but indicate consistencies with database sequences.

We used two approaches to determine the appropriateness of GV’s scoring system. GV scores for 10 000 randomly selected Swissprot genes were significantly higher than GV scores for 10 000 randomly selected TrEMBL genes (Supplementary Fig. S4). Similarly, 73–90% of recently updated gene models from four eukaryotic genomes had higher GV scores than older versions (Supplementary Table S1; Supplementary Fig. S5). Both results are consistent with GV appropriately quantifying gene prediction improvements due to manual curation or improved gene prediction technologies. Lower GV scores for some gene predictions could be due the reference databases containing sequences of low-quality, new automated predictions introducing new errors and scores being noisy for queries with few BLAST hits.

## 5 Future work

GV was developed with a plug-in system for adding validation approaches. We plan to extend GV with improved orthology detection, additional validation approaches (e.g. codon usage, explicit RNAseq support) and improved statistics (e.g. evidence-weighting based on phylogenetic and database-quality information). In its current form, GV already can save large amounts of time for biologists working with newly obtained gene predictions.

## Supplementary Material

Supplementary Data
