# Supplementary material for: GeneValidator: identify problems with protein-coding gene predictions
Source: Bioinformatics. 2016 Jan 18;32(10):1559–61. doi: 10.1093/bioinformatics/btw015 (PMC4866521; doi:10.1093/bioinformatics/btw015)
Supplement: Supplementary Data [file supp_32_10_1559__index.html]

GeneValidator: identify problems with protein-coding gene predictions — GeneValidator: identify problems with protein-coding gene predictions — Supplementary Data 

# GeneValidator: identify problems with protein-coding gene predictions

## Supplementary Data

files

- Supplementary Data - pdf file
