# Supplementary material for: GeneValidator: identify problems with protein-coding gene predictions
Source: Bioinformatics. 2016 Jan 18;32(10):1559–61. doi: 10.1093/bioinformatics/btw015 (PMC4866521; doi:10.1093/bioinformatics/btw015)
Supplement: Supplementary Data [file supp_btw015_Dragan_et_al_GeneValidator_suppl_info_reviewed.pdf]

Database selection

Lower GV scores for some gene predictions could be due to the reference databases containing sequences of low-quality, new automated predictions introducing new errors, and scores being noisy for queries with few BLAST hits. Therefore, GV results for a gene prediction strongly depend on the quality of the reference database, on the number of similar sequences which were identified and how similar they are to the query gene. Choosing databases thus requires considering multiple tradeoffs. For example, SwissProt includes only high-quality gene predictions that were manually curated by expert curators, but only from few species. The same can be true for known curated gene models in NCBI RefSeq, sequences in gene-family-specific databases as well as private unpublished databases that may result from manual curation and/or molecular approaches. However, for many gene predictions (e.g., for genes from smaller gene families, or from taxa distant to those for which extensive curation was performed), additional databases are generally needed. Uniprot/TrEMBL and Genbank NR contain many more sequences, but these are largely automatic predictions and thus likely include major errors. In some cases it can thus be worthwhile to run GV multiple times using different databases or combinations of databases.

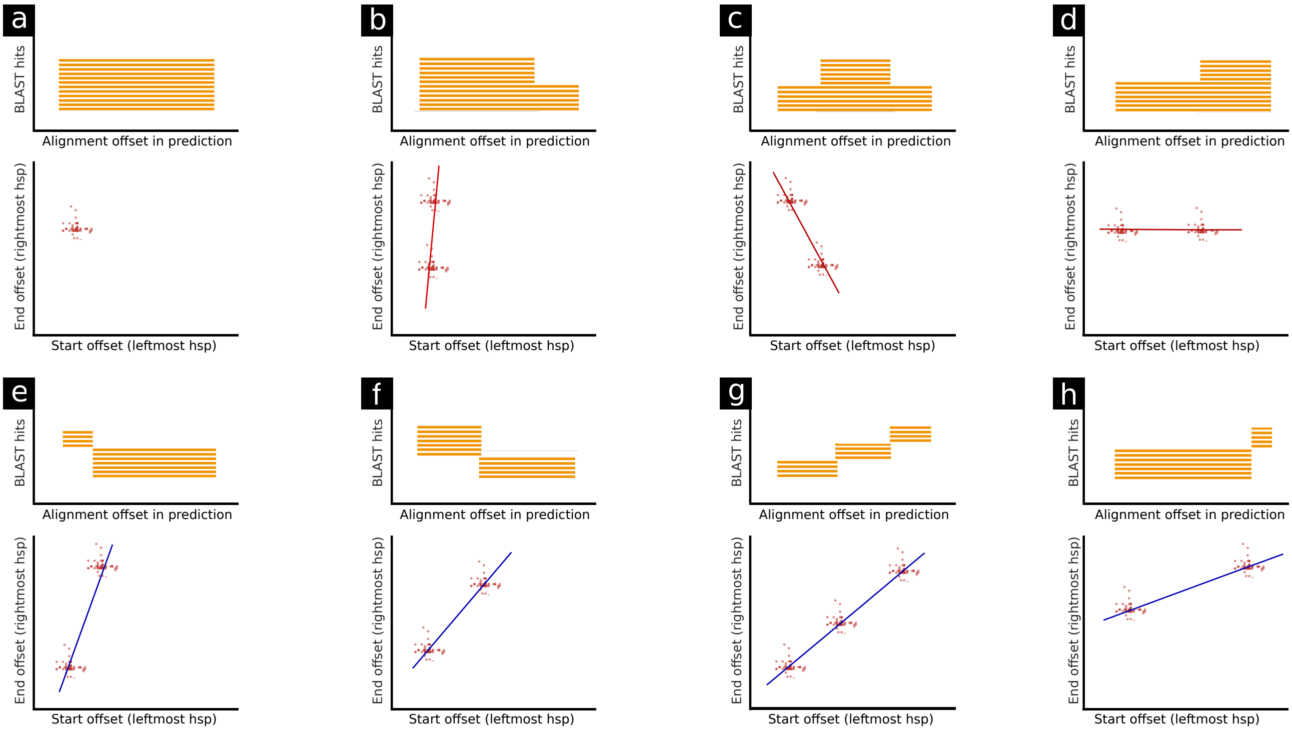

Figure 1. Examples of BLAST overview graphs (first row) and HSP offset coordinate graphs (second row) for eight query sequences. Query sequences used for a), b), c) and d) each represented single genes. In these cases HSP coordinates are distributed a) unimodally or have regression slopes (red lines) that are b) vertical, c) negative, and d) horizontal. In contrast, query sequences used for e), f), g), and h) each result from merging multiple unrelated genes. In these cases, HSP coordinate distribution regression slopes are between 0.4 and 1.2 (blue lines).

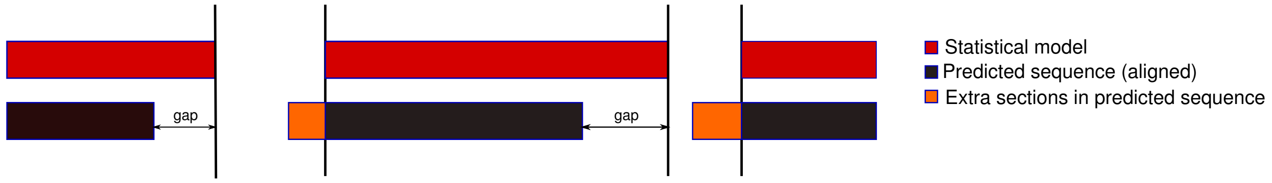

Figure 2. Illustration of differences that can be observed between a position specific scoring matrix profile (statistical model derived from multiple alignment of the ten strongest BLAST hits) and a predicted query sequence.

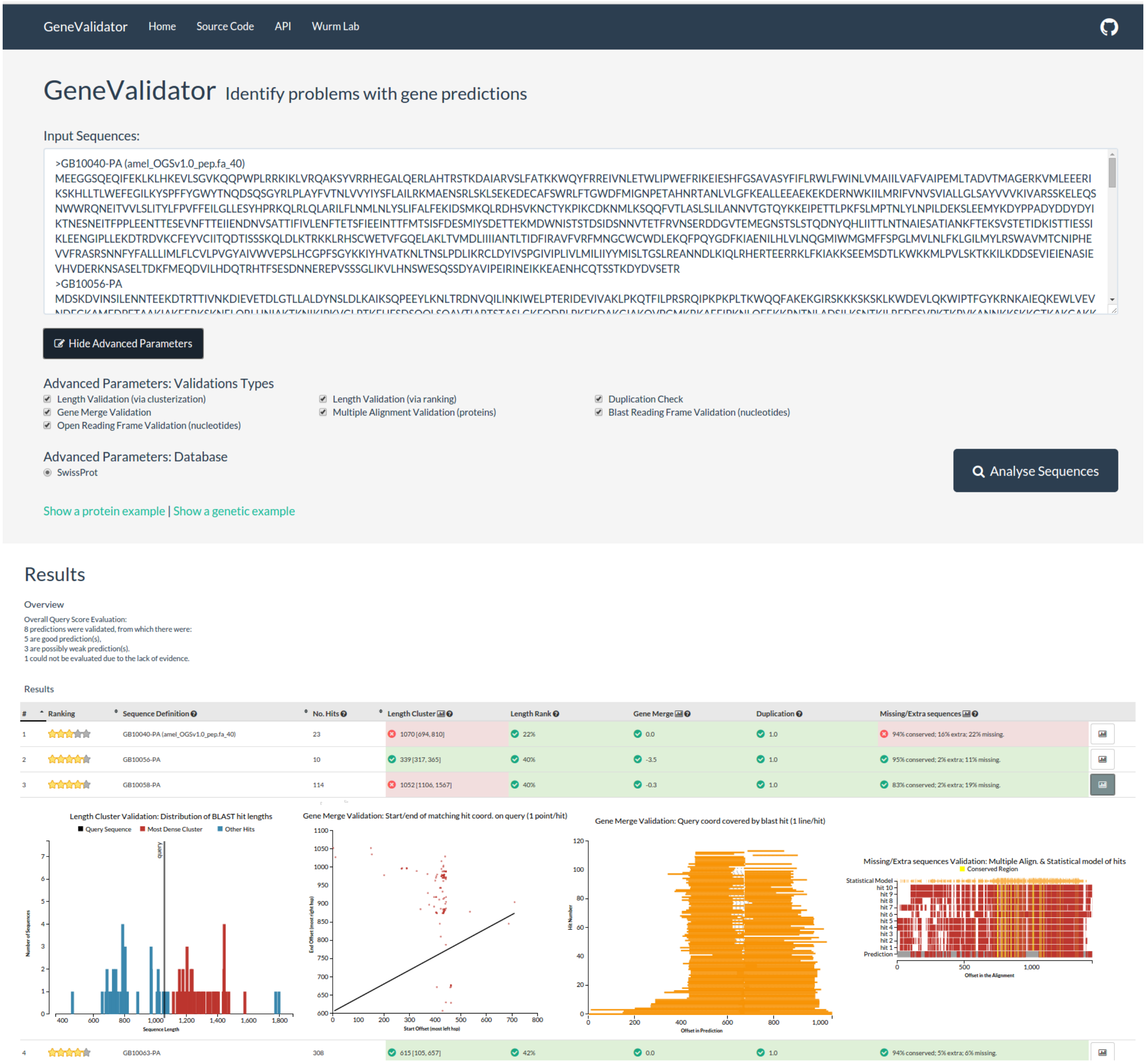

Figure 3. Screenshots of GeneValidator web app query interface and results overview as accessible at http://genevalidator.sbcs.qmul.ac.uk.

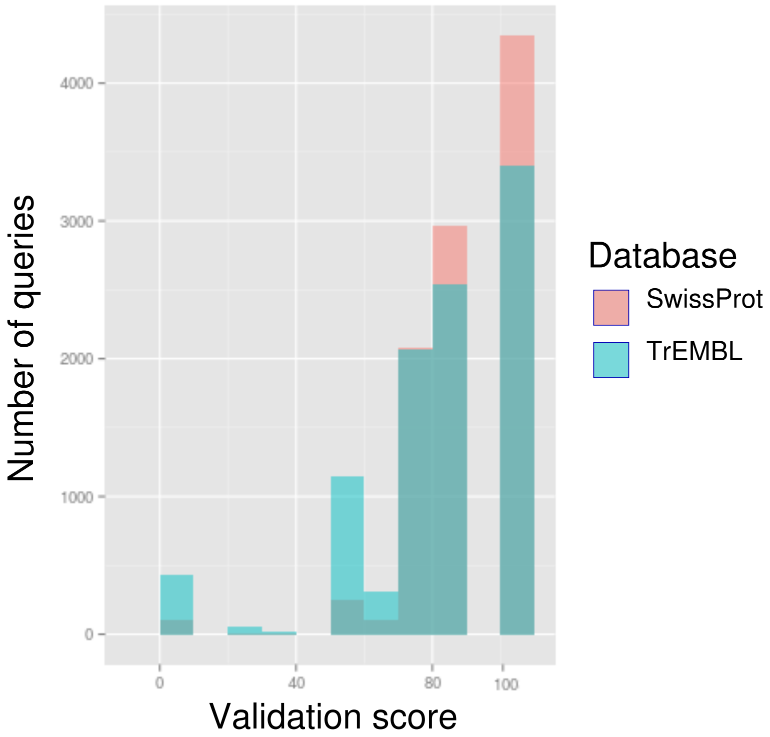

Figure 4. Overall score distribution (from 0 to 100) of GV validations against the NR database for 10,000 random genes from Uniprot/Swiss-Prot in red (mean: 88.31) and 10,000 random genes from Uniprot/TrEMBL in blue (mean: 80.02). The higher scores of Swiss-Prot proteins (Wilcox test: $p \leq 2.2 \times 10^{-16}$) are consistent with GeneValidator more highly scoring gene models curated by expert humans than automatic predictions (The UniProt Consortium 2014).

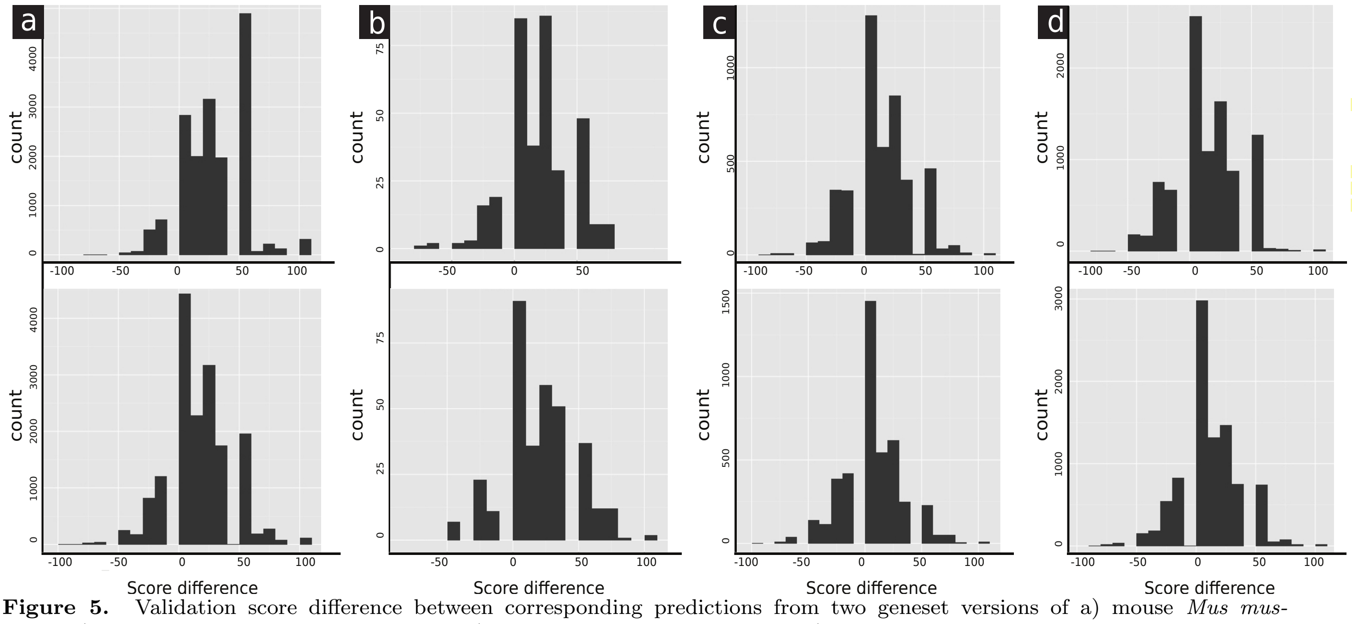

Figure 5. Validation score difference between corresponding predictions from two geneset versions of a) mouse *Mus musculus*, b) argentine ant *Linepithema humile*, c) honeybee *Apis mellifera* and d) zebrafish *Danio rerio*, validated against NR database (first row) and against Swiss-Prot database (second row). Correspondence of the genes between pairs of geneset versions was found by reciprocal blast. Genes with identical scores between genesets were not reported. Code for this analysis is at https://github.com/wurmlab/genevalidator/tree/genevalidator-validator.

**Genome**	**Gene set: number of genes**	**Different GV scores; Number (%) improved**
Mus musculus (Flicek *et al.*, 2014)	v2003: 32,910<br>v2013: 51,437	14,186;<br>12,809 (90%)
Linepithema humile (Smith *et al.*, 2011)	v1.1: 16,177<br>v1.2: 16,226	262;<br>219 (83%)
Apis mellifera (Weinstock *et al.*, 2006) (Elsik *et al.*, 2014)	v1.0: 10,157<br>v3.2: 15,314	3,300;<br>2,415 (73%)
Danio rerio (Flicek *et al.*, 2014)	v2009: 28,717<br>v2013: 42,555	6,748;<br>4,962 (74%)

Table 1. Comparison of GV results from old and newer versions of official gene sets from four genome projects.

References

Elsik, C. G. *et al.* (2014). Finding the missing honey bee genes: Lessons learned from a genome upgrade. *BMC Genomics*, **15**, 86.

Flicek, P. *et al.* (2014). Ensembl 2014. *Nucleic Acids Res*, **42**, D749–55.

Smith, C. D. *et al.* (2011). Draft genome of the globally widespread and invasive Argentine ant (*Linepithema humile*). *PNAS*, **108**, 5673–8.

Weinstock, G. M. *et al.* (2006). Insights into social insects from the genome of the honeybee *Apis mellifera*. *Nature*, **443**, 931–49.
